# Supplementary material for: Impaired Pharmacokinetics of Amiodarone under Veno-Venous Extracorporeal Membrane Oxygenation: From Bench to Bedside
Source: Pharmaceutics. 2022 Apr 30;14(5):974. doi: 10.3390/pharmaceutics14050974 (PMC9147299; doi:10.3390/pharmaceutics14050974)

Supplemental data:

Figure S1: Diagnostic plots of final model

A. Observed concentrations vs predicted concentrations of populations

B. Observed concentrations vs individual predicted concentrations

C. Individual weighted residues (IWRES) in time

D. Individual weighted residues (IWRES) vs observed concentrations (Cc)

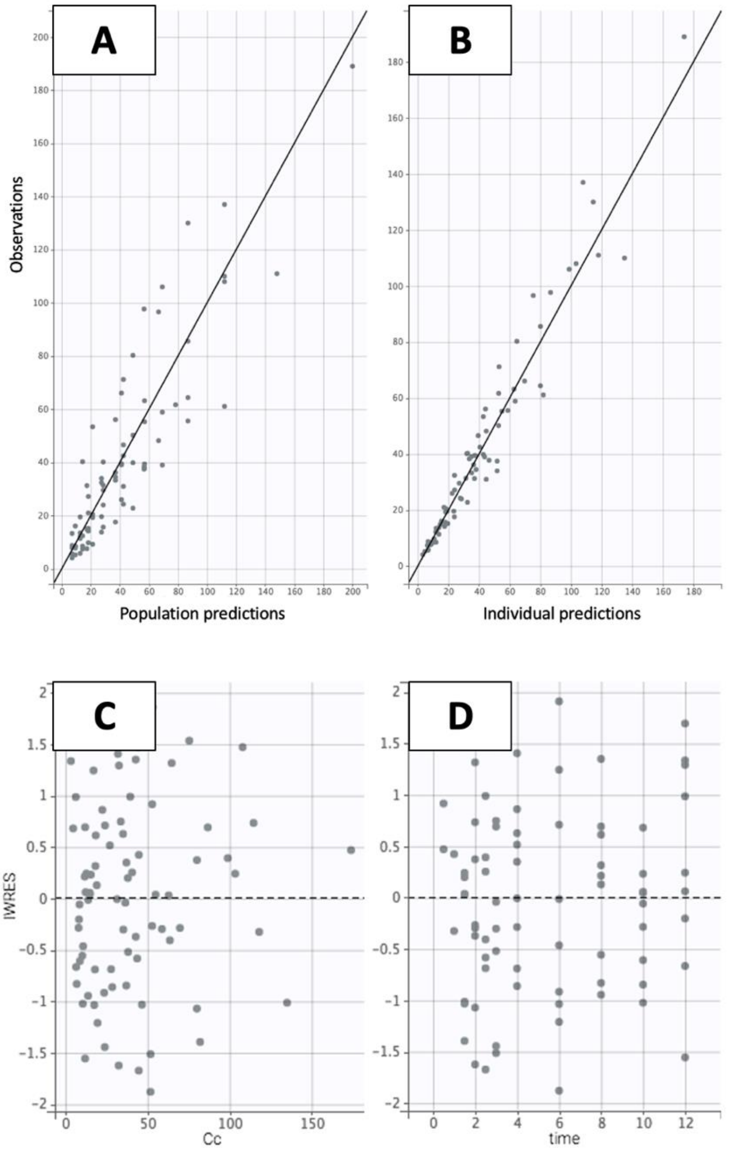

Supplement: Supplementary file 1 [file pharmaceutics-14-00974-s001.zip › pharmaceutics-1689542-supplementary.pdf]
